# Matrix Metalloproteinase-9 Inhibition Improves Proliferation and Engraftment of Myogenic Cells in Dystrophic Muscle of mdx Mice

**DOI:** 10.1371/journal.pone.0072121

**Published:** 2013-08-15

**Authors:** Sajedah M. Hindi, Jonghyun Shin, Yuji Ogura, Hong Li, Ashok Kumar

**Affiliations:** Department of Anatomical Sciences and Neurobiology, University of Louisville School of Medicine, Louisville, Kentucky, United States of America; University of Minnesota Medical School, United States of America

## Abstract

Duchenne muscular dystrophy (DMD) caused by loss of cytoskeletal protein dystrophin is a devastating disorder of skeletal muscle. Primary deficiency of dystrophin leads to several secondary pathological changes including fiber degeneration and regeneration, extracellular matrix breakdown, inflammation, and fibrosis. Matrix metalloproteinases (MMPs) are a group of extracellular proteases that are involved in tissue remodeling, inflammation, and development of interstitial fibrosis in many disease states. We have recently reported that the inhibition of MMP-9 improves myopathy and augments myofiber regeneration in mdx mice (a mouse model of DMD). However, the mechanisms by which MMP-9 regulates disease progression in mdx mice remain less understood. In this report, we demonstrate that the inhibition of MMP-9 augments the proliferation of satellite cells in dystrophic muscle. MMP-9 inhibition also causes significant reduction in percentage of M1 macrophages with concomitant increase in the proportion of promyogenic M2 macrophages in mdx mice. Moreover, inhibition of MMP-9 increases the expression of Notch ligands and receptors, and Notch target genes in skeletal muscle of mdx mice. Furthermore, our results show that while MMP-9 inhibition augments the expression of components of canonical Wnt signaling, it reduces the expression of genes whose products are involved in activation of non-canonical Wnt signaling in mdx mice. Finally, the inhibition of MMP-9 was found to dramatically improve the engraftment of transplanted myoblasts in skeletal muscle of mdx mice. Collectively, our study suggests that the inhibition of MMP-9 is a promising approach to stimulate myofiber regeneration and improving engraftment of muscle progenitor cells in dystrophic muscle.

## Introduction

Duchenne muscular dystrophy (DMD) is caused by null mutations in the dystrophin gene that encodes a membrane-associated structural protein [1]–[3]. While the genetic basis of DMD has been known for more than 20 years, the pathophysiological mechanisms by which deficiency of dystrophin leads to muscle wasting in DMD remain less understood. Matrix metalloproteinases (MMPs) are a group of extracellular proteases that are involved in extracellular matrix (ECM) remodeling, inflammation, and development of interstitial fibrosis in many organs [4], [5]. Previous studies from our and other groups have shown that the abundance and activity of MMP-9 are increased in skeletal muscle of animal models of DMD [6]–[9]. Moreover, levels of MMP-9 are also increased in serum and muscle biopsies of patients with DMD and MMP-9 correlates with the severity of disease progression [10]. We have previously reported that the inhibition of MMP-9 using genetic or pharmacological approaches considerably improves skeletal muscle structure and function in both young and aged mdx mice [7], [11]–[13]. Of note are the findings that there is a marked increase in number of centronucleated and embryonic myosin heavy chain (eMyHC)-positive myofibers upon ablation of MMP-9 in adult mdx mice suggesting that inhibition of MMP-9 improves regeneration in dystrophic muscle [13]. However, the mechanisms by which MMP-9 regulates skeletal muscle regeneration in mdx mice remain unknown.

Macrophages are one of the most important cells of the immune system which infiltrate muscle tissue after injury. Macrophages can play both deleterious and beneficial roles in progression of myopathy in mdx mice [14], [15]. Macrophages have two subpopulations designated as M1 and M2 [14]. M1 macrophages, activated by proinflammatory Th1 cytokines such as IFN-γ, are known to promote fiber injury through production of nitric oxide, inflammatory cytokines, or direct phagocytosis [7], [14], [16]. By contrast, IL-4 and IL-13 promote M2 macrophage phenotype that is characterized by the production of anti-inflammatory cytokines such as IL-10 [14]. M2 macrophages make the dystrophic muscle less inflammatory and attenuate myofiber injury in mdx mice [15]. Furthermore, recent studies have provided evidence that alternatively activated M2 macrophages augment muscle regeneration through stimulating the proliferation of satellite cells in mdx mice [14], [17]. Therefore, modulating the relative proportion of M1 and M2 macrophages can influence the course and severity of disease progression in DMD.

Notch and Wnt signaling pathways are involved in proliferation and differentiation of satellite cells in adult myofiber upon injury [18]–[21]. Notch signaling is required not only for the proliferation of satellite cells but also for maintaining the satellite cell pool for following rounds of muscle regeneration [22], [23]. Activation of Notch signaling occurs within a day post-injury [21]. Replicative senescence observed in aged muscle has been associated with diminished activation of Notch pathway [21], [24]. Forced activation of Notch signaling in injured aged muscle restored muscle regenerative potential in a manner similar to that seen in aged/young parabiotic pairings [21], [24]. Moreover, Wnt signaling also plays a major role especially during later stages of muscle regeneration [25]. Wnt signaling promotes myogenic differentiation both *in vivo* and *in vitro* [20]. When Wnt signaling was inhibited 3–4 days post-injury in wild-type mice, it led to reduced satellite cell differentiation and impairment in muscle regeneration [25]. However, it remains unknown whether MMP-9 modulates the activation of Notch and Wnt signaling in skeletal muscle of mdx mice.

Recent advancement in stem cell research has provided strong evidence that muscle specific stem cell (i.e. satellite cells) is one of the most important approaches for the introduction of functional dystrophin protein in patients with DMD [26]–[30]. However, isolation of satellite cells and their introduction into muscle tissue typically results in poor engraftment and loss of much of the stem cell population, resulting in little repair of the diseased or injured tissue [28]. Besides immune response, the migration and engraftment of satellite cells can also be greatly influenced by the presence of excessive fibrosis and extracellular matrix deposition in muscle tissues [28]. Strategies that reduce secondary changes prior to satellite cell transplantation can potentially improve the functional engraftment of satellite cells in dystrophic muscles [31]. Indeed, the attenuation of inflammation in muscle tissue has been found to improve myoblast survival in cellular therapy in models of DMD [28], [31]. Although inhibition of MMP-9 improves myofiber regeneration and reduces fibrosis in 8-week old mdx mice [13], it remains unknown whether the inhibition of MMP-9 can also augment the engraftment of transplanted muscle progenitor cells in dystrophic muscle of mdx mice.

In the present study, we investigated the mechanisms by which inhibition of MMP-9 improves regeneration in skeletal muscle of mdx mice. We have previously reported that heterozygous (i.e. mdx;Mmp9^+/−^) or homozygous (i.e. mdx;Mmp9^−/−^) deletion of *Mmp9* gene significantly improves myopathy in 8-week old mdx mice [13]. One of the unique aspects of our study was that the deletion of a single allele of *Mmp9* gene was sufficient to reduce protein levels of MMP-9 by ∼80% in skeletal muscle of mdx mice. Our published study also provided evidence that although both heterozygous and homozygous deletion of *Mmp9* gene attenuates myopathy, the improvement observed in mdx;Mmp9^+/−^ mice is better compared to that of mdx;Mmp9^−/−^ mice [13]. Furthermore, the formation of new myofibers was increased more dramatically in mdx;Mmp9^+/−^ mice compared to mdx;Mmp9^−/−^ mice indicating that a small amount of MMP-9 may be beneficial whereas excessive amounts of MMP-9 exacerbates myopathy in mdx mice [13]. It is also notable that partial inhibition of MMP-9 is clinically relevant because it is not possible to completely block the activity of any protein using pharmacological drugs as has been the case with knockout models. For these reasons, in the present study, we have employed only mdx;Mmp9^+/+^ and mdx;Mmp9^+/−^ mice to delineate the mechanisms by which inhibition of MMP-9 improves myofiber regeneration in mdx mice. Our results demonstrate that the inhibition of MMP-9 increases the number of satellite cells in skeletal muscle of mdx mice. Inhibition of MMP-9 also increases the proportion of anti-inflammatory M2 macrophages and stimulates Notch and canonical Wnt signaling in mdx mice. Finally, our experiments demonstrate that MMP-9 inhibition dramatically improves the engraftment of myoblasts in dystrophic muscle of mdx mice. These results provide initial evidence that blocking the activity of MMP-9 is an important approach to improving myofiber regeneration and for satellite cell-based therapy for patients with DMD.

## Materials and Methods

### Mice

Control (strain: C57BL/10 ScSnJ), mdx (strain: C57BL/10 ScSn DMD^mdx^), *Mmp9*-knockout (strain: FVB.Cg-*Mmp9* ^tm1Tvu^/J), and mT/mG (strain: Gt(ROSA)26Sor^tm4(ACTB-tdTomato,-EGFP)Luo^/J) mice were purchased from Jackson Laboratory (Bar Harbor, ME, USA). *Mmp9*-knockout mice were first crossed with C57BL10/ScSn mice for seven generations and then with mdx mice to generate littermate wild-type, mdx;Mmp9^+/+^ and mdx;Mmp9^+/−^ mice as previously described [13]. All genotypes were determined by PCR analysis from tail DNA. Mice were housed in the animal facility of the University of Louisville under conventional conditions with constant temperature and humidity and fed a standard diet. All experiments with animals were approved by the Institutional Animal Care and Use Committee of the University of Louisville.

### Primary Myoblast Cultures

Primary myoblasts were isolated from hind limb muscle of mTmG mice that express membrane-targeted tandem dimer Tomato (mT, a red fluorescent protein) and cultured following similar protocol as previously described [11]. Briefly, hind limb skeletal muscles from mice were aseptically isolated, minced into coarse slurry, and enzymatically digested at 37°C for 1 h by adding 200 IU/ml collagenase I (Worthington, cat # LS004196) and 0.1% pronase (EMD Chemicals). Digested slurry was filtered through a 70 µm filter, and spun and isolated cells were resuspended and cultured initially in F-10 medium (containing 20% FBS and supplemented with 10 ng/ml basic fibroblast growth factor) and then in F-10 plus Dulbecco’s Modified Eagle Medium (DMEM) (1∶1 ratio) based culture medium supplemented with 15% FBS on culture dishes coated with 10% matrigel (BD Biosciences).

### Myoblast Transplantation in Skeletal Muscle of mdx Mice

Muscle regeneration in mdx;Mmp9^+/+^ and mdx;Mmp9^+/−^ mice was induced by injecting cardiotoxin (CTX, 50 µl of 10 µM solution, Sigma) into the mid-belly of tibial anterior (TA) muscles 24 h before cell transplantation. About 5×10^5^ mTmG myoblasts were transplanted into the mid-belly of CTX-injected TA muscle. Muscles were harvested 28 days post-myoblast transplanation and analyzed for mT fluorescence and dystrophin expression.

### Histology and Morphometric Analysis

Skeletal muscle tissues were removed, frozen in isopentane cooled in liquid nitrogen, and sectioned in a microtome cryostat. For the assessment of tissue morphology, 10-µm-thick transverse sections of each muscle were stained with H&E, and staining was visualized on a microscope (Eclipse TE 2000-U), a digital camera (Digital Sight DS-Fi1), and NIS Elements BR 3.00 software (all from Nikon). The images were stored as JPEG files, and image levels were equally adjusted using Photoshop CS2 software (Adobe). Percentage of centronucleated fibers was determined by counting the number of fibers with nuclei in center divided by total number of fibers in each section. The extent of fibrosis in muscle cryosections was determined using a Sirius red dye staining kit following a protocol suggested by manufacturer (American Master Tech).

### Indirect Immunofluorescence

For immunohistochemistry study, muscle sections were blocked in 1% bovine serum albumin in PBS for 1 h, and incubated with anti-Pax7 (1∶20, Developmental Studies Hybridoma Bank, University of Iowa, Iowa City, IA), anti-E-MyHC (1∶150, Developmental Studies Hybridoma Bank, University of Iowa, Iowa City, IA), or anti-dystrophin (1∶50, Abcam) in blocking solution at 4°C overnight under humidified conditions. The sections were washed briefly with PBS before incubation with Alexa Fluor® 488 or 594-conjugated secondary antibody (1∶3000, Invitrogen) for 1 h at room temperature and then washed 3 times for 5 minutes with PBS. The slides were mounted using fluorescence medium (Vector Laboratories) and visualized at room temperature on Nikon Eclipse TE 2000-U microscope (Nikon), a digital camera (Nikon Digital Sight DS*-*Fi1), and Nikon NIS Elements BR 3.00 software (Nikon). Image levels were equally adjusted using Abode Photoshop CS2 software (Adobe). Necrotic fibers in muscle cryosections were identified by immunostaining with Cy3-labelled goat anti-mouse IgG (1∶3000, Invitrogen).

### Western Blotting

Quantitative estimation of specific protein was done by Western blot using a method as previously described [32]. Briefly, skeletal muscle tissues were washed with phosphate-buffered saline (PBS) and homogenized in lysis buffer A [50 mM Tris-Cl (pH 8.0), 200 mM NaCl, 50 mM NaF, 1 mM dithiotheritol (DTT), 1 mM sodium orthovanadate, 0.3% IGEPAL, and protease inhibitors]. Approximately, 100 µg protein was resolved on each lane on 12% SDS-PAGE, electrotransferred onto nitrocellulose membrane and probed using anti-Wnt3a (1∶500, Millipore), anti-Axin2 (1∶1000, Abcam), MMP-9 (1∶500, R&D Systems), and anti-tubulin (1∶2000, Cell Signaling Inc) and detected by chemiluminescence.

### RNA Isolation and Quantitative Real-time PCR (QRT-PCR)

RNA isolation and QRT-PCR were performed using a method as previously described [33]. The sequence of the primers is described in Table S1.

### Fluorescence Activated Cell Sorting (FACS)

Activated satellite cells and M1 and M2c macrophages were analyzed by FACS as previously described [34]. Approximately 2×10^6^ cells were incubated in DMEM (supplemented with 2% FBS and 25 mM 4-(2-hydroxyethyl)-1-piperazineethanesulfonic acid)) and dead cells (positive for Propidium iodide staining) which were around ∼1% were excluded from all FACS analysis. For satellite cell quantification from heterogeneous cell population, cells were immunostained with antibodies against, CD45, CD31, and Ter-119 for negative selection (all PE conjugated, eBiosciences), and with α7-integrin (MBL International) for positive selection. A tandem conjugate of R-PE (Alexa 647, Molecular Probes) was used as a secondary antibody against α7-integrin. Macrophages were quantified from heterogeneous cell population by selection of F4/80^+^ (PerCP Cy5.5-conjugated, eBiosciences) cells against negative selection by CD56/Sca-1 and Ter-119 (all PE-conjugated, eBiosciences). From F4/80^+^ cells, CD11c^+^ (APC-conjugated, eBiosciences) M1 and CD206^+^ (FITC-conjugated, Biolegend) M2 macrophages were isolated. FACS analysis was performed on a C6 Accuri cytometer equipped with three lasers. The output data was processed and plots were prepared using FCS Express 4 RUO software (De Novo Software).

### Statistical Analysis

Results are expressed as mean ± standard deviation (SD). Statistical analysis used Student’s t-test (two tailed) to compare quantitative data populations with normal distribution and equal variance. A value of P<0.05 was considered statistically significant unless otherwise specified.

## Results

### Effects of Inhibition of MMP-9 on Initial Myofiber Injury in mdx Mice

The levels of MMP-9 are higher in skeletal muscle of mdx mice in both prenecrotic and necrotic states [13]. We first sought to determine whether inhibition of MMP-9 affects skeletal muscle structure at prenecrotic stages of mdx mice. In mdx mice, muscle injury starts at around 2.5 weeks followed by peak necrotic phase in combination with inflammation between 3–4 weeks of age [35], [36]. Regeneration starts around the age of 6 weeks and continues while alternating with ongoing degeneration until 12 weeks of age [37], [38]. Therefore, to understand the role of MMP-9 at prenecrotic state, we used 2-week old littermate mdx;Mmp9^+/+^, mdx;Mmp9^+/−^, and mdx;Mmp9^−/−^ mice. Gastrocnemius (GA) muscle was isolated from these mice and muscle sections made were processed for Hematoxylin and Eosin (H&E) and Picro-Sirius Red (for collagens) staining. As shown in Figure S1 (upper panel), no apparent difference in muscle structure was evident between mdx;Mmp9^+/+^, mdx;Mmp9^+/−^, and mdx;Mmp9^−/−^ mice. Furthermore, the level of collagen was comparable in skeletal muscles of these mice (Figure S1, middle panel). We also immunostained muscle sections for eMyHC protein. A negligible amount of myofibers (2–3 per section) stained positive for eMyHC. However, there was no significant difference in number of eMyHC positive fibers in GA muscle of mdx;Mmp9^+/+^, mdx;Mmp9^+/−^, and mdx;Mmp9^−/−^ mice (Figure S1, lower panel). These results suggest that inhibition of MMP-9 does not affect skeletal muscle structure in mdx mice at the prenecrotic stage.

**Figure 1:**
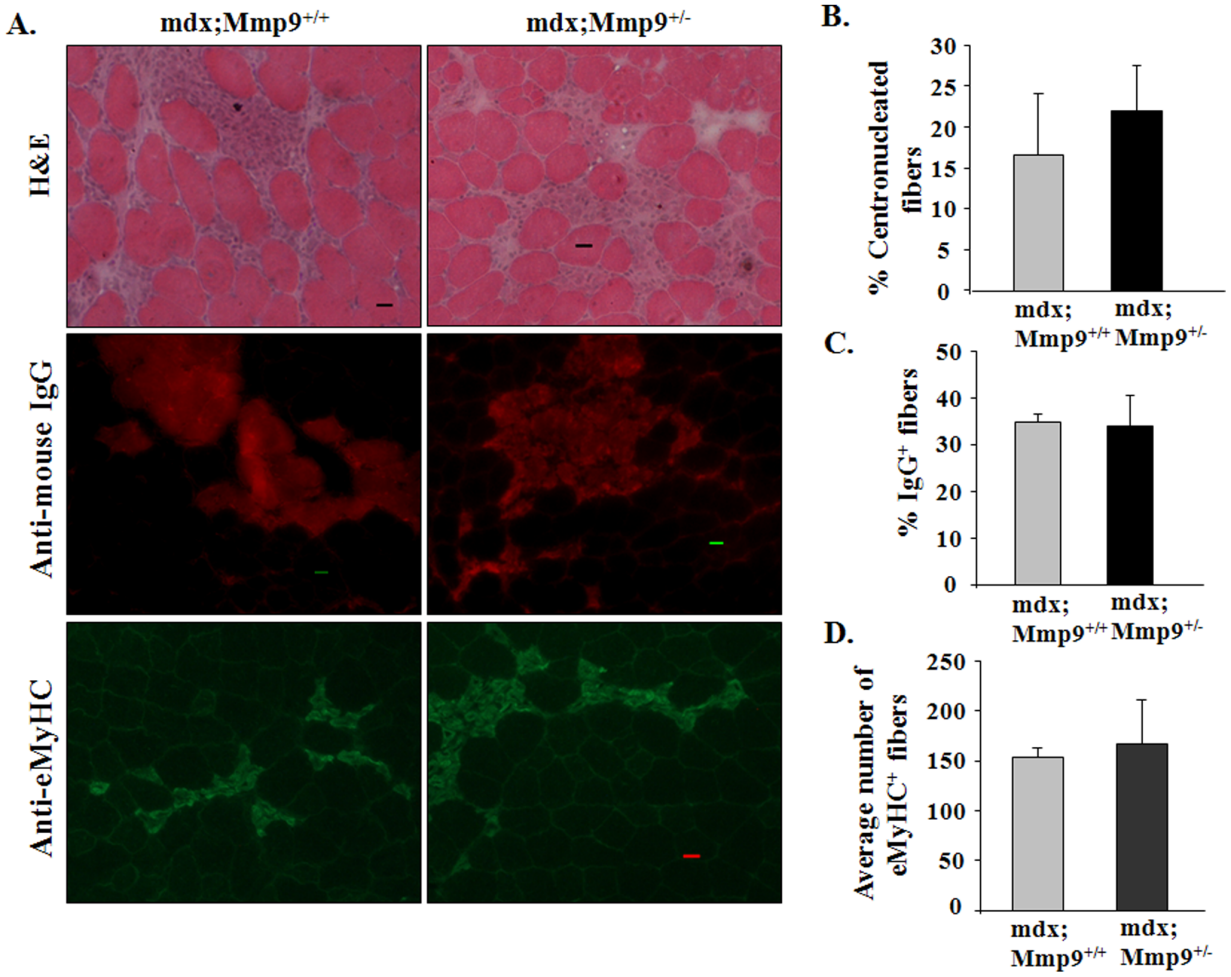
MMP-9 is not involved in the initial onset of muscle pathology in mdx mice. (**A**) Representative photomicrographs of GA muscle sections of 4-week old mdx;Mmp9^+/+^ and mdx;Mmp9^+/−^ mice displaying hallmarks of mdx pathology. Area under necrosis and centronucleated myofibers evaluated by H&E staining (top panel). Muscle sections were immunostained with Cy3-labeled goat anti-mouse IgG to detect permeable/damaged fibers (middle panel). Formation of new myofibers was assessed by staining with embryonic myosin heavy chain (eMyHC) antibody (lower panel). Scale bar: 20 µm. Quantification of (**B**) percentage of centronucleated fibers; (**C**) Cy3-labelled IgG filled fibers per field; and (**D**) number of eMyHC-positive fibers per field of mdx;Mmp9^+/+^ and mdx,MMP9^+/−^ mice. N = 4 in each group. Error bars represent SD.

We have previously reported that area under necrosis is significantly reduced in skeletal muscle of 8-week old mdx;Mmp9^+/−^ and mdx;Mmp9^−/−^ mice and it is filled with newly formed centronucleated fibers [13]. However, it is not clear whether the inhibition of MMP-9 also reduces initial wave of fiber necrosis in mdx mice. To address this issue, we analyzed skeletal muscle of mdx;Mmp9^+/+^ and mdx;Mmp9^+/−^ mice at the age of 4-week, where fiber necrosis is at peak. As shown in Figure 1, the area under necrosis was comparable in H&E-stained muscle sections from mdx;Mmp9^+/+^ and mdx;Mmp9^+/−^ mice. Furthermore, there was no difference in the number of centronucleated fibers between mdx;Mmp9^+/−^ and mdx;Mmp9^+/+^ mice (Figure 1A, 1B). As a measure of sarcolemmal injury, we performed IgG staining on muscle sections. The number of IgG-filled fibers was comparable in skeletal muscle of mdx;Mmp9^+/+^ and mdx;Mmp9^+/−^ mice (Figure 1A, 1C). We next performed immunostaining for eMyHC on muscle sections of mdx;Mmp9^+/+^ and mdx;Mmp9^+/−^ mice. The number of eMyHC-positive fibers in mdx;Mmp9^+/−^ was not significantly different from mdx;Mmp9^+/+^ mice (Figure 1A, 1D). Moreover, the proportion of F4/80^+^, M1, or M2 macrophages was comparable in dystrophic muscle of 4-week old mdx;Mmp9^+/−^ and mdx;Mmp9^−/−^ mice measured by FACS analysis (Figures S2A, S2B, S2C). Western blot analysis showed that heterozygous deletion of Mmp9 gene reduces MMP-9 protein levels by ∼50% in dystrophic muscle of mdx mice (Figure S2D). It is noteworthy that the number of macrophages and MMP-9 levels are dramatically reduced with concomitant improvement in muscle pathology at the age of 8-week in mdx;Mmp9^+/−^ mice compared to mdx;Mmp9^+/+^ mice [13]. Taken together, these results suggest that the inhibition of MMP-9 does not protect myofibers from initial injury. However, continued inhibition of MMP-9 may prevent further myofiber injury through diminishing secondary pathological changes and hastening regeneration of injured myofibers which are clearly evident at the age of 7–8 weeks [13].

### Inhibition of MMP-9 Improves the Number of Satellite Cells in Dystrophic Muscle of mdx Mice

**Figure 2:**
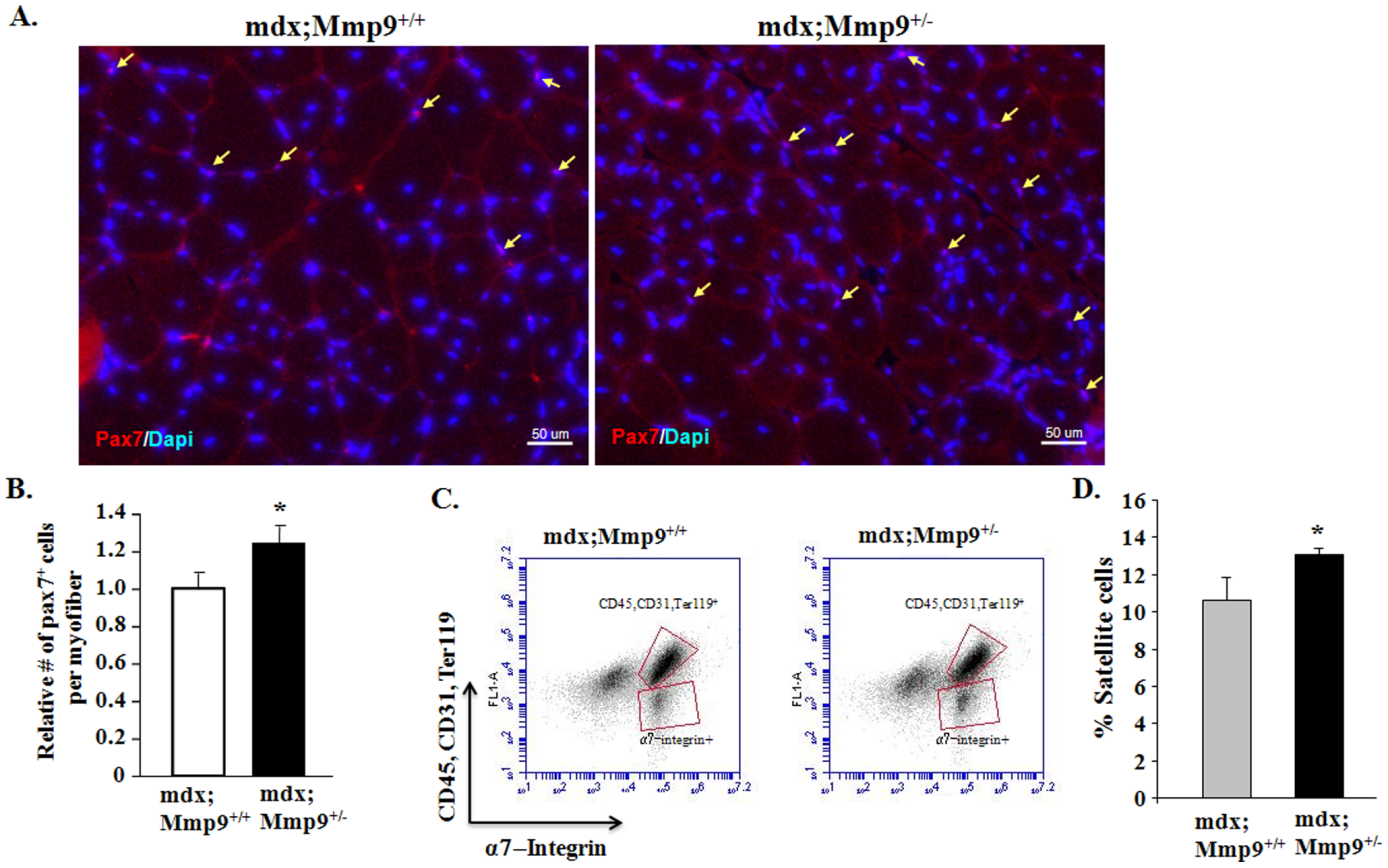
Inhibition of MMP-9 enhances satellite cell population in skeletal muscle of mdx mice. (**A**) Representative photomicrograph of TA muscle section from 8-week old mdx;Mmp9^+/+^ and mdx;Mmp9^+/−^ stained for Pax7 and DAPI. Arrows point to Pax7^+^ satellite cells. Scale bar: 50 µm. (**B**) Quantification of relative number of Pax7^+^ cells per myofiber in TA muscle sections from 8-week old mdx;Mmp9^+/+^ and mdx;Mmp9^+/−^ mice. (**C**) Representative FACS dot plots showing the percentage of satellite cells in GA muscle of 8-week old mdx;Mmp9^+/+^ and mdx;Mmp9^+/−^ mice. Negative selection antibodies (CD45, CD31, Ter119) are in upper box whereas positive selection antibody (α7-integrin) is in lower box. (**D**) Quantification of satellite cells by FACS in GA muscles of 8-week mdx;Mmp9^+/+^ and mdx;Mmp9^+/−^ mice. N = 4 in each group Error bars represent SD. *p<0.05 values significantly different from that of mdx;Mmp9^+/+^ mice.

We have previously reported that heterozygous or homozygous deletion of Mmp9 significantly improves myofiber regeneration in 8-week old mdx mice. In particular, the numbers of centronucleated myofibers and eMyHC-positive myofibers per unit area were significantly increased in mdx;Mmp9^+/−^ and mdx;Mmp9^−/−^ mice compared with mdx;Mmp9^+/+^ mice [13]. To understand the mechanisms by which MMP-9 inhibits skeletal muscle regeneration in mdx mice, we examined activation of satellite cells which are known to facilitate regeneration of injured muscle in both normal and disease conditions [39]. Pax7 is an important marker of both quiescent and activated satellite cells [22], [40]. We first performed immunostaining for Pax7 to evaluate satellite cells in skeletal muscle of mdx;Mmp9^+/+^ and mdx;Mmp9^+/−^ mice. Number of Pax7^+^ cells per myofiber was found to be significantly higher in tibial anterior (TA) muscle of 8-week old mdx;Mmp9^+/−^ compared to mdx;Mmp9^+/+^ mice (Figures 2A, 2B). Previous studies have found that a unique combination of cell surface markers (CD45^−^, CD31^−^, Ter119^−^, and α7- integrin^+^) identify satellite cells in adult mouse skeletal muscle and allow their direct quantification by fluorescence-activated cell sorting (FACS) technique [41]. To further evaluate whether MMP-9 affects the activation of satellite cells in skeletal muscle of mdx mice, we next performed FACS analysis. Gating strategy for quantification of satellite cells by FACS has been previously depicted [34]. Single cell suspension was made from gastrocnemius (GA) muscle and the proportion of satellite cells in cellular mixture was quantified using FACS technique. Consistent with immunohistochemistry results, proportion of satellite cells was found to be significantly higher in mdx;Mmp9^+/−^ mice compared to littermate mdx;Mmp9^+/+^ mice (Figures 2C, 2D). Taken together, these results demonstrate that the inhibition of MMP-9 improves satellite cell proliferation in dystrophic muscle of mdx mice.

### Inhibition of MMP-9 Suppresses M1 and Promotes M2 Macrophage Phenotype in Skeletal Muscle of mdx Mice

**Figure 3:**
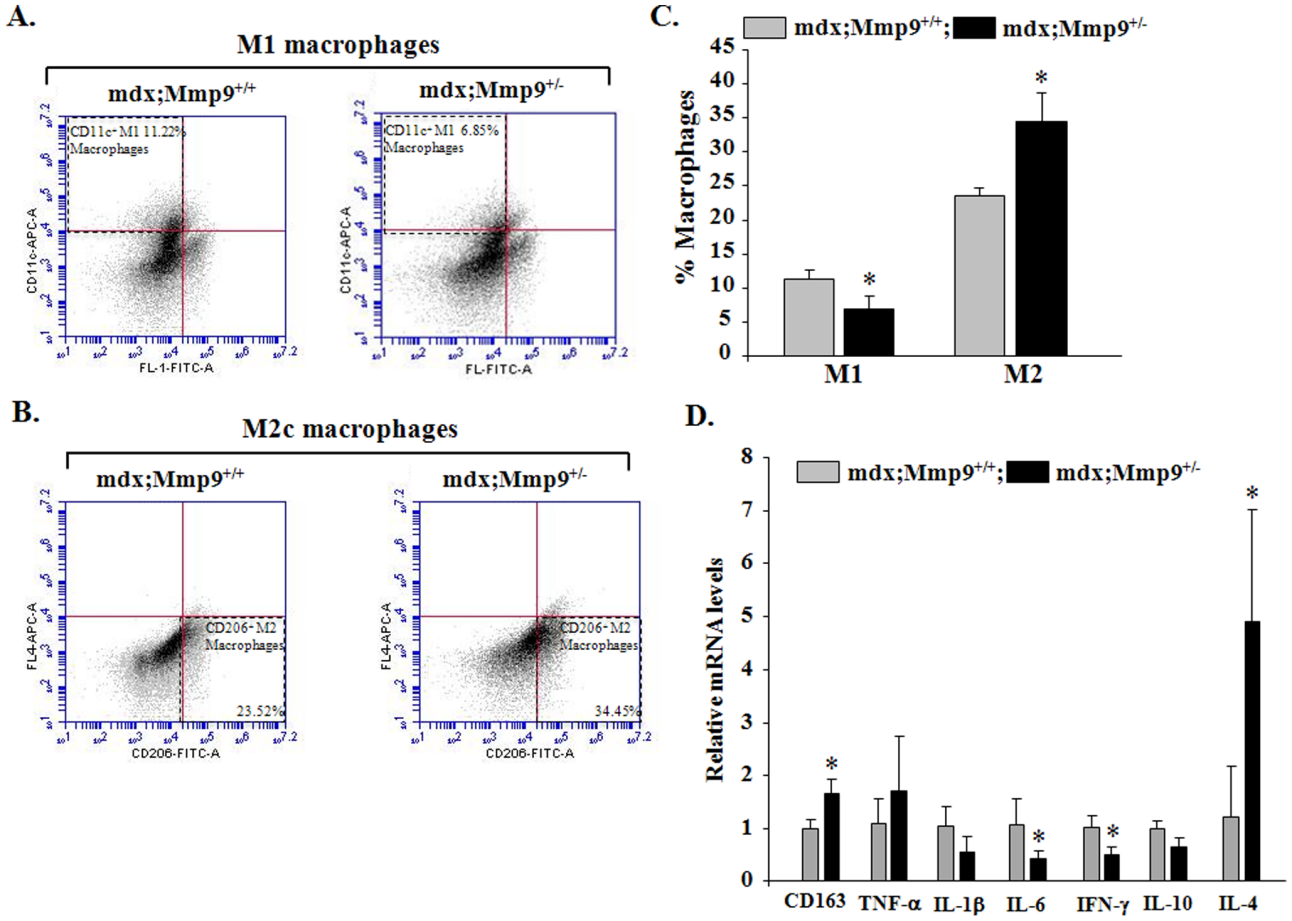
Inhibition of MMP-9 promotes M2 macrophage phenotype in skeletal muscle of mdx mice. GA muscle of 8-week old mdx;Mmp9^+/+^ and mdx;Mmp9^+/−^ mice were isolated and processed for FACS analysis for M1 and M2 macrophages in the F4/80^+^ population after gating out CD45 and Sca1 cells. (**A**) FACS dot plots displaying the percentage of CD11c^+^ M1-macrophages (upper left quadrant). (**B**) Representative dot plots for the percentage of CD206^+^ M2 macrophages (lower right quadrant). (**C**) Quantification of relative proportion of M1 and M2 macrophages by FACS technique in GA muscle of mdx;Mmp9^+/+^ and mdx;Mmp9^+/−^ mice. (**D**) Fold change in relative mRNA levels of CD163, TNF-α, IL-1β, IL-6, IFN-γ, Il-10, and IL-4 measured by QRT-PCR assay in GA muscle of 8-week old mdx;Mmp9^+/+^ and mdx;Mmp9^+/−^ mice. N = 3 or 4 in each group. Error bars represent SD. *p<0.05, values significantly different from mdx;Mmp9^+/+^ mice.

M1 and M2 macrophages play critical roles in fiber degeneration and regeneration, respectively [14]. It has been reported that proinflammatory M1 macrophages dominate the necrotic phase of pathology in mdx mice contributing to muscle damage while a shift towards an anti-inflammatory M2 phenotype prevails at the regenerative stage facilitating repair [15], [17]. We examined the relative amounts of M1 and M2 macrophages in F4/80^+^ population in 8-week old mdx;Mmp9^+/−^ and their littermate mdx;Mmp9^+/+^ mice by FACS technique. As shown in Figure 3A and 3C, the proportion of M1 macrophages (CD11c^+^) was significantly higher in skeletal muscle of 8-week old mdx;Mmp9^+/+^ (∼11.22%) compared to mdx;Mmp9^+/−^ (∼6.85%). By contrast, proportion of CD206^+^ (a marker of M2a and M2c) macrophages was significantly elevated in dystrophic muscle of mdx;Mmp9^+/−^ mice (∼34.45%) compared to littermate mdx;Mmp9^+/+^ (∼23.52%) mice (Figure 3B, 3C).

Previous studies have shown that inflammatory cytokines TNF-α, IN-1β, IL-6, and IFN-γ inhibit whereas anti-inflammatory cytokines IL-4 and IL-10 promote a shift towards M2 phenotype in macrophages [14], [42]. By performing QRT-PCR, we investigated whether the inhibition of MMP-9 also affects the levels of key cytokines which influence macrophage phenotype in skeletal muscle of mdx mice. As shown in Figure 3D, the transcript levels of IFN-γ and IL-6 were significantly reduced whereas transcript levels of IL-4 were significantly increased which is consistent with increased population of M2 macrophages in skeletal muscle of mdx;Mmp9^+/−^ mice compared to mdx;Mmp9^+/+^ mice. However, transcript levels of IL-10 were not significantly different despite a significant increase in M2c macrophages evident by significant increase in mRNA levels of CD163 (a marker for M2c macrophages) in skeletal muscle of mdx;Mmp9^+/−^ compared to mdx;Mmp9^+/+^ mice (Figure 3D). Furthermore, there was no significant difference in the mRNA levels of TNF-α and IL-1β in dystrophic muscle of 8-week old mdx;Mmp9^+/+^ and mdx;Mmp9^+/−^ mice (Figure 3D).

### Inhibition of MMP-9 Improves Notch Signaling in Dystrophic Muscle of mdx Mice

**Figure 4:**
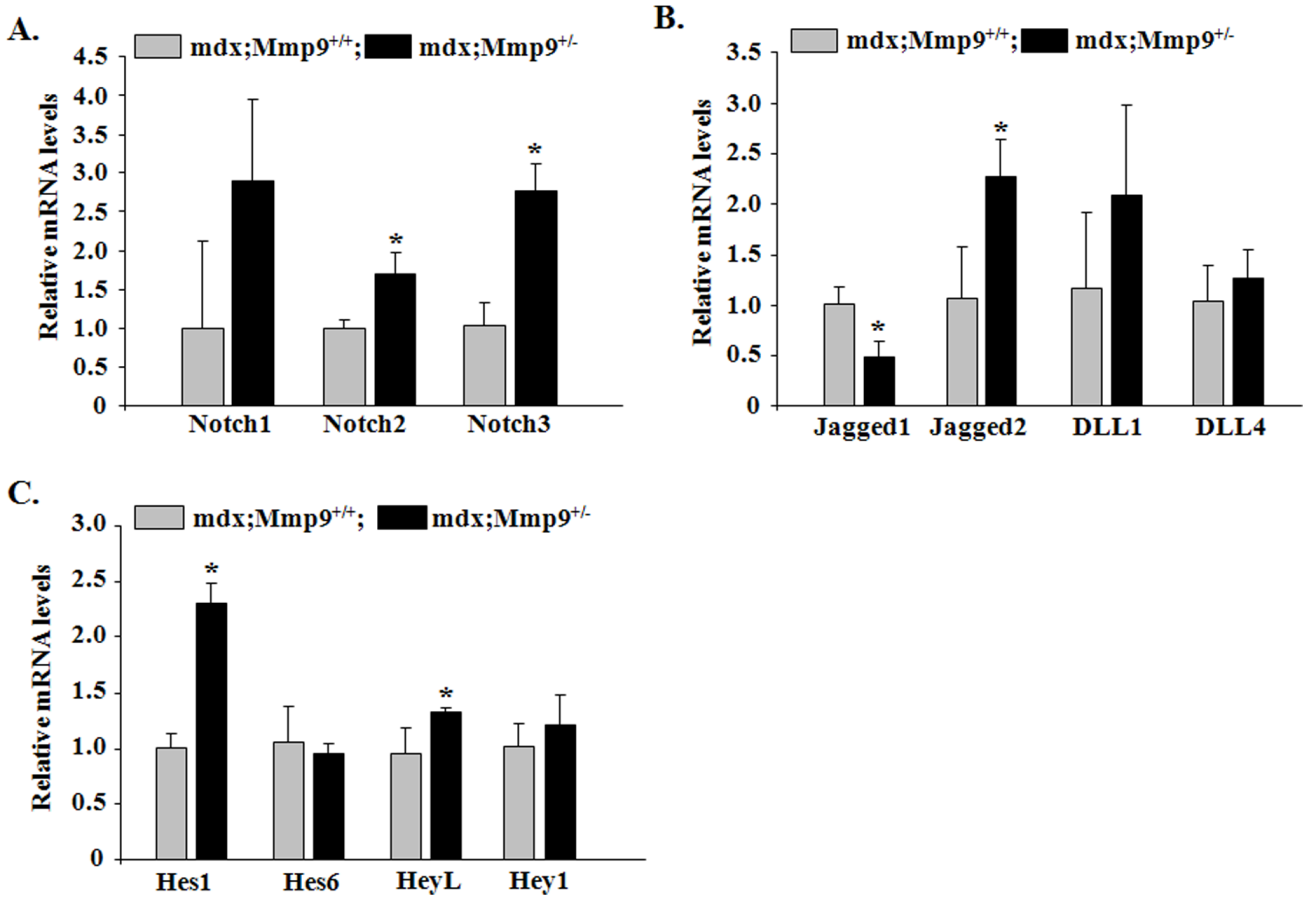
Inhibition of MMP-9 increases Notch signaling in skeletal muscle of mdx mice. GA muscles were isolated from 8-week old mdx;Mmp9^+/+^ and mdx;Mmp9^+/−^ mice and processed for QRT-PCR analysis. Fold change in expression of (**A**) Notch receptors- Notch1, Notch2 and Notch3; (**B**) Notch ligands- Jagged1, Jagged2, DLL1 and DLL4; and (**C**) Notch target genes- Hes1, Hes6, HeyL, and Hey1. N = 3–4 in each group. Error bars represent SD. *p<0.05, values significantly different from littermate mdx;Mmp9^+/+^ mice.

The Notch signaling pathway is one of the most documented pathways known to be involved in the activation and commitment of muscle progenitor cells to myogenic lineage during embryonic and postnatal myogenesis [21]. In addition, Notch signaling is required for maintaining the satellite cell pool for following rounds of muscle regeneration [22], [23]. Given the observed improvement in number of satellite cells in mdx;Mmp9^+/−^ compared to mdx;Mmp9^+/+^ mice, we next sought to investigate whether the inhibition of MMP-9 affects Notch signaling in skeletal muscle of mdx mice. To study the activation of Notch signaling, we measured transcript levels of Notch ligands (Jagged1, Jagged2, DLL1 and DLL4), receptors (Notch1, Notch2 and Notch3) and target genes (Hes1, Hes6 and HeyL) by QRT-PCR assay. Interestingly, transcript levels of Notch2, Notch3, Jagged2, Hes1 and HeyL were found to be significantly increased whereas levels of Jagged1 reduced in skeletal muscle of 8-week old mdx;Mmp9^+/−^ compared to littermate mdx;Mmp9^+/+^ mice (Figures 4A, 4B, 4C). These results suggest that Notch signaling is increased skeletal muscle of mdx mice upon inhibition of MMP-9.

### Inhibition of MMP9 Differentially Regulates Canonical and Non-canonical Wnt Pathways in Dystrophic mdx Muscle

**Figure 5:**
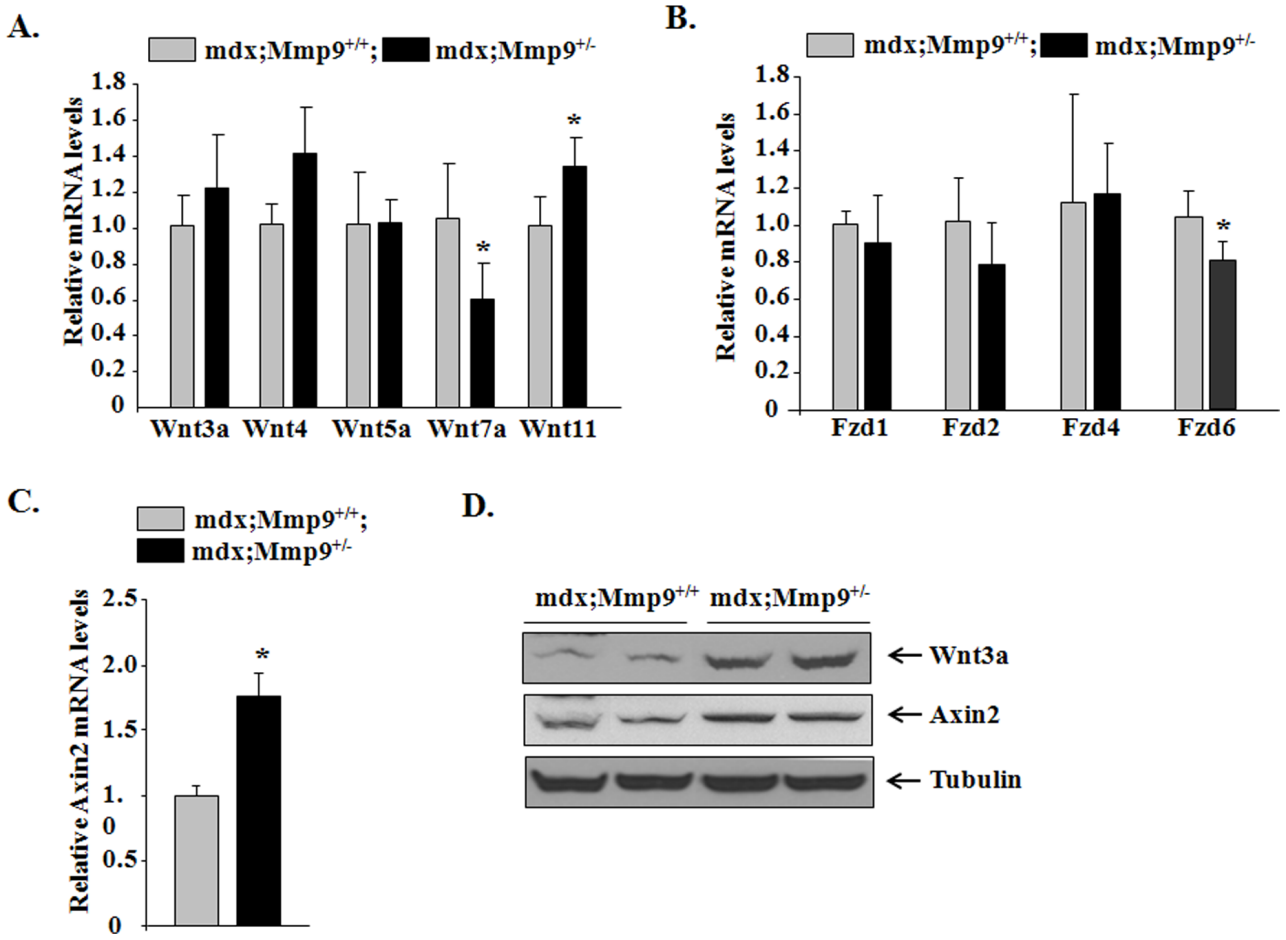
Inhibition of MMP9 improves canonical Wnt pathway but suppresses non-canonical Wnt signaling. GA muscles isolated from 8-week old mdx;Mmp9^+/+^ and mdx;Mmp9^+/−^ mice and processed for QRT-PCR or Western blotting. (**A**) Relative mRNA levels of Wnt3a, Wnt4, Wnt5a, Wnt7a and Wnt11. (**B**) Relative mRNA levels Frizzled (Fzd)1, Fzd2, Fzd4 and Fzd6. (**C**) Relative mRNA levels of Axin2. N = 3 (for Western) and N = 3 (for QRT-PCR) in each group. (**D**) Representative immunoblots showing Wnt3a and Axin2 and unrelated protein tubulin in GA muscle. Error bars represent SD. *p<0.01, values significantly different from littermate mdx;Mmp9^+/+^ mice.

Successful muscle regeneration requires adequate activation of satellite cells followed by their differentiation and fusion to form mature myofibers [39]. Our previous study has demonstrated that 8-week old mdx;Mmp9^+/−^ mice possess an augmented regenerative ability evident by increased e-MyHC expression and formation of new fibers [13] potentially driven by an anti-inflammatory and Notch-rich environment. The β-catenin-dependent canonical Wnt pathway is involved in the later stages of muscle regeneration in injured muscle of wild-type mice [25]. Blocking Wnt activity 3d post-injury led to defective muscle regeneration characterized by persistence in proliferating myoblasts and interstitial infiltrates void of mature muscle fibers which illustrate the importance of canonical Wnt signaling in muscle differentiation and/or fusion [25], [43]. By contrast, non-canonical Wnt pathway has been shown to activate planar cell polarity and Akt signaling leading to muscle hypertrophy [21]. To test the possible role of MMP-9 in regulation of canonical and non-canonical Wnt signaling in myofibers of mdx mice, we studied the expression levels of specific components of both these pathways. Transcript levels of canonical Wnt pathway components, Wnt11, Axin2, were found to be significantly induced in skeletal muscle of mdx;Mmp9^+/−^ muscle compared to littermate mdx;Mmp9^+/+^ mice (Figure 5A, 5B, 5C). Although mRNA levels of Wnt3a were higher in mdx;Mmp9^+/−^ mice compared to mdx;Mmp9^+/+^, they were not statistically different (Figure 5A). By contrast, our Western blot analysis showed that protein levels of Wnt3a as well as Axin2 (a target gene of Wnt pathway) were considerably increased in skeletal muscle of mdx;Mmp9^+/−^ mice compared to mdx;Mmp9^+/+^ mice (Figure 5D). These results indicate that Wnt3a may also be expressed in other tissues and the increased amounts of Wnt3a in skeletal muscle of mdx;Mmp9^+/−^ may be attributed to its increased levels in the circulation. Alternatively, inhibition of MMP-9 may improve the stability of Wnt3a protein in dystrophic muscle. More interestingly, we found that the transcript levels of some of the members of non-canonical Wnt pathway, namely Wnt7a and frizzled 6 (Fzd6), were significantly reduced in skeletal muscle of mdx;Mmp9^+/−^ compared to mdx;Mmp9^+/+^ mice (Figure 5A, 5B). These results suggest that MMP-9 differentially regulates the activation of canonical and non-canonical arms of Wnt signaling.

### Inhibition of MMP9 Improves Myoblast Engraftment in Dystrophic Muscle of mdx Mice

**Figure 6:**
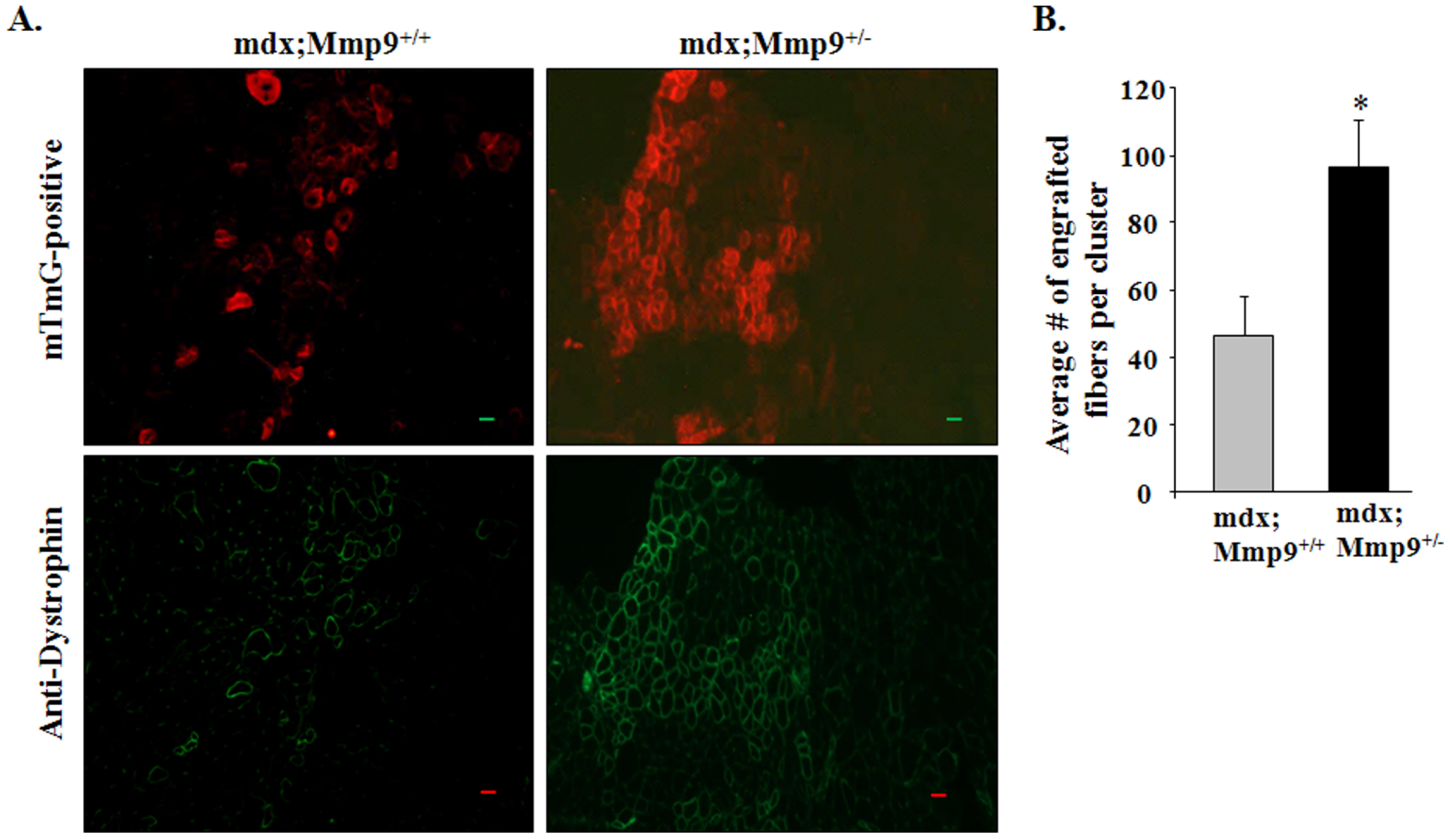
Inhibition of MMP-9 improves engraftment of transplanted cells in skeletal muscle of mdx mice. After 24 h of cardiotoxin injection, TA muscle of mdx;Mmp9^+/+^ and mdx;Mmp9^+/−^ were injected with 5×10^5^ myoblasts that were prepared from mTmG mice. After 28 days, TA muscle was isolated and engraftment of transplanted myoblasts into dystrophic muscle was analyzed directly or by staining with dystrophin antibody. (**A**) Representative images show mT protein florescence (top panel) and dystrophin (lower panel) in TA muscle section of mdx;Mmp9^+/+^ and mdx;Mmp9^+/−^ mice. Scale bar: 50 µm. (**B**) Quantification of the extent of myoblast engraftment. Average number of engrafted myofibers per cluster (area where implanted myoblasts are stained positive) is reported. N = 8 in each group. Error bars represent SD. *p<0.05 values significantly different from mdx;Mmp9^+/+^ mice.

Since inhibition of MMP-9 improves satellite cell proliferation and myofiber formation in mdx mice, we next sought to determine whether suppression of MMP-9 levels can also improve engraftment of transplanted myoblasts into dystrophic muscle of mdx mice. TA muscle of 8-week old littermate mdx;Mmp9^+/+^ and mdx;Mmp9^+/−^ mice were given intramuscular injection of cardiotoxin for 24 h followed by injection of cultured myoblasts from mT/mG mice (expressing mT red fluorescence protein). After 4 weeks, the TA muscle were isolated and visualized directly for mT florescence (red) or after co-staining for dystrophin (green) using a fluorescent microscope. Interestingly, transplanted TA muscle of mdx; Mmp9^+/−^ displayed ∼45% increase in mT/mG and dystrophin-positive fibers compared to mdx;Mmp9^+/+^ mice (Figure 6A, 6B). Engrafted myofibers in transplanted mdx;Mmp9^+/−^ were closely associated in large patches and extended beyond transplantation site compared to a disbursed distribution of engrafted fibers in mdx;Mmp9^+/+^ that where more localized to transplantation points (Figure 6A). These results demonstrate that in addition to facilitating endogenous satellite cell activation, MMP-9 inhibition modulates the muscle microenvironment to support implanted myoblasts engraftment into dystrophic muscle.

## Discussion

Skeletal muscle has remarkable ability to regenerate in response to injury by activation of undifferentiated myogenic precursor cells (known as satellite cells) [39], a mechanism known to be impaired in DMD patients [44]–[46]. It has been suggested that the reduced myofiber regeneration in DMD may result from the exhaustion of satellite cells due to excessive cycles of fiber degeneration and regeneration [45], [47], [48]. However, no decline in muscle regeneration was evident in mdx mice when the muscle was subjected to repeated cycles of degeneration and regeneration [49] suggesting that other factors may also contribute to impairment of muscle regeneration in DMD. The loss of regenerative capacity could also result from a progressive increase in muscle interstitial fibrosis since this would prevent the availability of growth factors and migration of myogenic cells required for muscle repair/regeneration [48], [50], [51]. Indeed, it has been reported that the addition of collagen I suppresses the differentiation of cultured satellites cells into myotubes [52]. Elevated amounts of proinflammatory cytokines that inhibit myoblast differentiation could also be a reason for reduced skeletal muscle regeneration in dystrophic muscles [53], [54]. Furthermore, since basement membrane (BM) is essential for myofiber regeneration and formation of neuromuscular junctions [55]–[58], extensive degradation of BM commonly observed in dystrophic muscles may also interfere with the ability of skeletal muscle to regenerate.

Excessive accumulation of MMPs has been observed in settings of muscle injury including that of DMD and known to exacerbate myopathy [7], [13], [59], [60]. Our previous findings show that the inhibition of MMP-9 improves skeletal muscle structure and function in 8-week [13] as well as 1-year-old mdx mice [11]. Moreover, inhibition of MMP-9 prevents fibrosis in skeletal and cardiac muscle of mdx mice [12], [13]. In the present study, we demonstrate that the beneficial effects achieved by inhibition of MMP-9 is a result of improved satellite cell activation and muscle formation potentially due to the activation of Notch and canonical Wnt signaling pathways. Our systematic analysis of disease progression illustrates that MMP-9 may have minimal role in the initial onset of muscle pathology evident by comparable display of disease manifestation in myofibers of mdx.;Mmp9^+/+^ and mdx;Mmp9^+/−^ at the age of 2 weeks (Figure S1) and 4 weeks (Figures 1). While we found reduced necrosis and inflammation at the age of 8-week, there was no significant difference in the level of muscle injury (Figure 1) and number of infiltrating macrophages (Figure 2S) in dystrophic muscle of mdx;Mmp9^+/+^ and mdx;Mmp9^+/−^ mice at the age of 4-week. However, based on these results, we cannot completely rule out the possibility that MMP-9 is involved in fiber necrosis in mdx mice especially at later stages of disease progression. It is noteworthy that MMP-9 functions by multiple mechanisms which include the activation of latent proinflammatory cytokines and chemokines, exaggerating inflammatory cells response, degradation of extracellular matrix components such as collagen IV and laminin, and proteolysis of β-dystroglycan. Most of these MMP-9-mediated responses are diminished in dystrophic muscle of mdx;Mmp9^+/−^ and mdx;Mmp9^−/−^ mice at the age of 8-week [13]. Based on these findings, we are tempted to speculate that the initial muscle injury that occurs due to sarcolemmal instability leads to the accumulation of inflammatory cells including macrophages in dystrophic muscle with no to minimal role of MMP-9. However, macrophages are the major source for the production of various inflammatory molecules including MMP-9. Indeed, we have previously reported that macrophages secrete MMP-9 in dystrophic muscle of mdx mice [13]. Furthermore, the number of macrophages and the activation of proinflammatory transcription factors nuclear factor-kappa B (NF-κB) and activator protein-1 (AP-1) are significantly reduced in dystrophic muscle of 8-week old mdx;Mmp9^+/−^ and mdx;Mmp9^−/−^ mice compared to mdx;Mmp9^+/+^ mice suggesting that there is a positive feedback mechanism between MMP-9 levels and inflammatory response in dystrophic muscle of mdx mice [13]. Partial or complete ablation of MMP-9 may reduce further myofiber injury and diminish other secondary changes providing a potential explanation for reduced fiber necrosis and improvement in muscle structure observed at the age of 8-week in mdx;Mmp9^+/−^ and mdx;Mmp9^−/−^ mice. Moreover, it is also possible that due to significant improvement in muscle regenerative capacity, the necrotic fibers are rapidly replaced by newly formed myofibers in mdx;Mmp9^+/−^ and mdx;Mmp9^−/−^ mice.

We have previously reported that inhibition of MMP-9 improves muscle regeneration in mdx mice [13]. The results of this study further support this premise because number of satellite cells is significantly increased in skeletal muscle of 8-week old mdx;Mmp9^+/−^ compared to mdx;Mmp9^+/+^ mice (Figure 2). Furthermore, inhibition of MMP-9 dramatically improved myoblast engraftment and subsequent dystrophin expression in skeletal muscle of mdx mice (Figure 6).

While precise temporal activation of different signaling pathways define the stages of muscle regeneration in injured wild-type muscle [25], [39], , these signaling boundaries seem to diffuse into one another in regenerating muscle of DMD pathology as a result of the continuous overlapping cycles of myofiber degeneration and regeneration. Although a timely orchestrated switch from Notch to canonical Wnt signaling pathway demarcates the transition from the inflammatory and proliferative phase to the regenerative and pro-muscle formation phase in normal injured muscle [25], the activation of these pathways may follow a different pattern in regenerating myofibers of mdx mice. Our findings reveal simultaneous activation of both pathways with a significant improvement in Notch and canonical Wnt signaling in mdx;Mmp9^+/−^ compared to mdx;Mmp9^+/+^ mice (Figures 4 and 5) which may be responsible for increased proliferation and differentiation of satellite cells leading to improved muscle formation. Increased activation of Notch signaling observed in skeletal muscle of mdx;Mmp9^+/−^ mice is in agreement with a previous report of a direct negative regulation between Notch2 and MMP-9 in human gastric carcinoma cells [62]. Notch2 amongst other components of Notch signaling is significantly upregulated in mdx;Mmp9^+/−^ compared to mdx;Mmp9^+/+^ mice (Figure 4A, 4B ).

Contrary to the canonical Wnt pathway, we found that non-canonical Wnt signaling is suppressed upon inhibition of MMP-9 evident by diminished expression of Wnt7a ligand and Frizzled 6 receptor (Figure 5A, 5B). These observations are in somewhat contradiction with a recently published report regarding the role of Wnt7a in amelioration of myopathy in mdx [63]. However, they support our previously published findings where we found that transgenic overexpression of MMP-9 causes fiber hypertrophy in wild-type mice in an Akt-dependent manner [11]. Furthermore, ablation of MMP-9 in mdx mice reduced fiber hypertrophy and blunted phosphorylation of Akt kinase [11]. These findings are supported by previously published reports where a decrease in activity of MAPKs and their downstream target activator protein-1 (AP-1) transcription factor has been reported in response to inhibition of MMPs in myofibers of mdx mice [7], [13]. MAPKs and subsequently AP-1 have been implicated downstream of non-canonical Wnt pathway [64] which is inhibited in skeletal muscle of mdx;Mmp9^+/−^ mice (Figure 5). It is noteworthy that while the overexpression of Wnt7a ameliorates myopathy [63] and our results show that the pro-regenerative phenotype observed as a result of MMP-9 inhibition is accompanied by a reduction in the non-canonical Wnt pathway, these findings do not necessarily oppose each other because activation of Wnt7a has been shown to cause muscle hypertrophy in mdx mice [63] whereas our studies have revealed that the inhibition of MMP-9 has no preservative effect against muscle injury but acts by expediting muscle regeneration. Therefore, the differential roles observed for Wnt7a in both studies may be a result of modulation of different parameters of mdx pathology.

MMP-9 activity in injured wild-type muscle has been shown to coincide with the onset of inflammation and is required for muscle cell proliferation as it persists until inflammation subsides facilitating muscle repair [59]. However, the inflammatory process in normal regenerating muscle is limited to a specific time point after which inflammation subsides and pro-regenerative cues dominate allowing for muscle formation [14]. Since skeletal muscle of mdx mice are subjected to continuous trauma, the proinflammatory phase is prolonged if not persistent leading to sustained activation of MMP-9. In accordance, we have found that inhibition of MMP-9 favors an anti-inflammatory environment characterized by decreased pro-inflammatory M1 macrophages (Figure 3A, 3C) and increased levels of anti-inflammatory and pro-regenerative CD206^+^ M2 macrophages (Figure 3B, 3C). Additionally, we found that the levels of inflammatory cytokines IFN-γ and IL-6 were reduced whereas the levels of IL- 4 were upregulated in response to MMP-9 inhibition. Taken together, our results suggest that the inhibition of MMP-9 promotes muscle regeneration through countering inflammatory processes leading to enhanced muscle formation. Even though the biased shift towards an M2 macrophage phenotype may be a result of the improved overall muscle environment, it is notable that notch signaling has been implicated in M2 polarization in a context-dependent manner. IL-4 stimulated M2 macrophage phenotype was shown to be associated with increased Notch expression in settings of inflammation and angiogenesis suggesting that Notch activity may mediate the function of M2 macrophages [65]. Similarly, it is also possible that M2 macrophages lead to increased activation of Notch signaling culminating in improved satellite cell proliferation in dystrophic muscle of mdx;Mmp9^+/−^ mice.

In summary, our results demonstrate that elevated levels of MMP-9 negatively regulate satellite cell activation and myofiber regeneration in mdx mice. Inhibition of MMP-9 improves the activation of endogenous satellite cells and facilitates exogenous myoblast engraftment and consequent expression of dystrophin in myofibers of mdx mice. Clinical trials utilizing signaling pathways such as Notch as a mean to expand myoblast prior to transplantation have been successful in achieving more functional engraftment but are still limited by the ability of cells to migrate after entering a dystrophic environment [66]. Given that inhibition of MMP-9 was sufficient to facilitate muscle cell survival through cooperative activation of multiple signaling pathways including Notch and Wnt, pharmacological inhibition of MMP-9 can be an important approach to improve satellite cell proliferation, migration, and subsequent gene expression inside the diseased muscle after transplantation.

## Supporting Information

Figure S1 **Effect of ablation of MMP-9 on skeletal muscle of prenecrotic mdx mice.** Representative photomicrographs show that heterozygous or homozygous deletion of Mmp9 gene does not affect muscle structure (top panel), fibrosis (middle panel), or regeneration (bottom panel) in gastrocnemius muscle of mdx mice at the age of 2 weeks (prenecrotic).
(TIF)

Figure S2 **Effect of heterozygous deletion of** ***Mmp9*** **gene on macrophage content and protein levels of MMP-9 in 4-week old mdx mice.** GA muscle of 4-week old mdx;Mmp9^+/+^ and mdx;Mmp9^+/−^ mice were isolated and processed for FACS analysis for F4/80^+^, CD11c^+^ (M1) and CD206^+^ (M2) macrophages after gating out CD45^+^ and Sca1^+^ cells. M1 and M2 macrophages were quantified in F4/80+ macrophage population. Bar diagrams presented here show (A) total F4/80^+^ macrophages, (B) M1 macrophages, and (C) M2 macrophages. Error bars represent SD. (D) Representative immunoblots showing MMP-9 and unrelated protein tubulin in GA muscle of 4-week old wild-type, mdx;Mmp9^+/+^, and mdx;Mmp9^+/−^ mice. Black lines indicate that intervening lanes have been spliced out.
(TIF)

Table S1 **Sequence of the primers used for QRT-PCR assay.**
(DOCX)
