# Supplementary figures and images for: Matrix Metalloproteinase-9 Inhibition Improves Proliferation and Engraftment of Myogenic Cells in Dystrophic Muscle of mdx Mice
Source: PLoS One. 2013 Aug 15;8(8):e72121. doi: 10.1371/journal.pone.0072121 (PMC3744489; doi:10.1371/journal.pone.0072121)

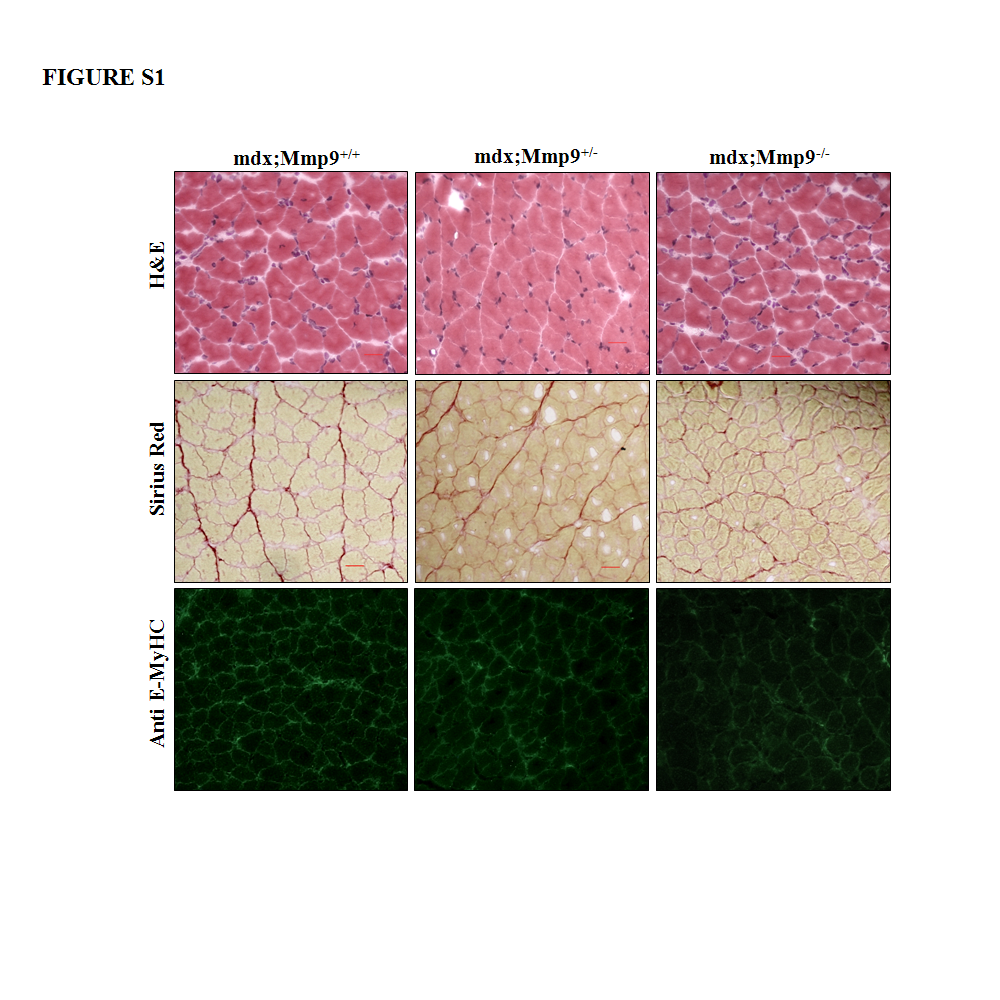

Supplement: Figure S1 — Effect of ablation of MMP-9 on skeletal muscle of prenecrotic mdx mice. Representative photomicrographs show that heterozygous or homozygous deletion of Mmp9 gene does not affect muscle structure (top panel), fibrosis (middle panel), or regeneration (bottom panel) in gastrocnemius muscle of mdx mice at the age of 2 weeks (prenecrotic). (TIF) [file pone.0072121.s001.tif]

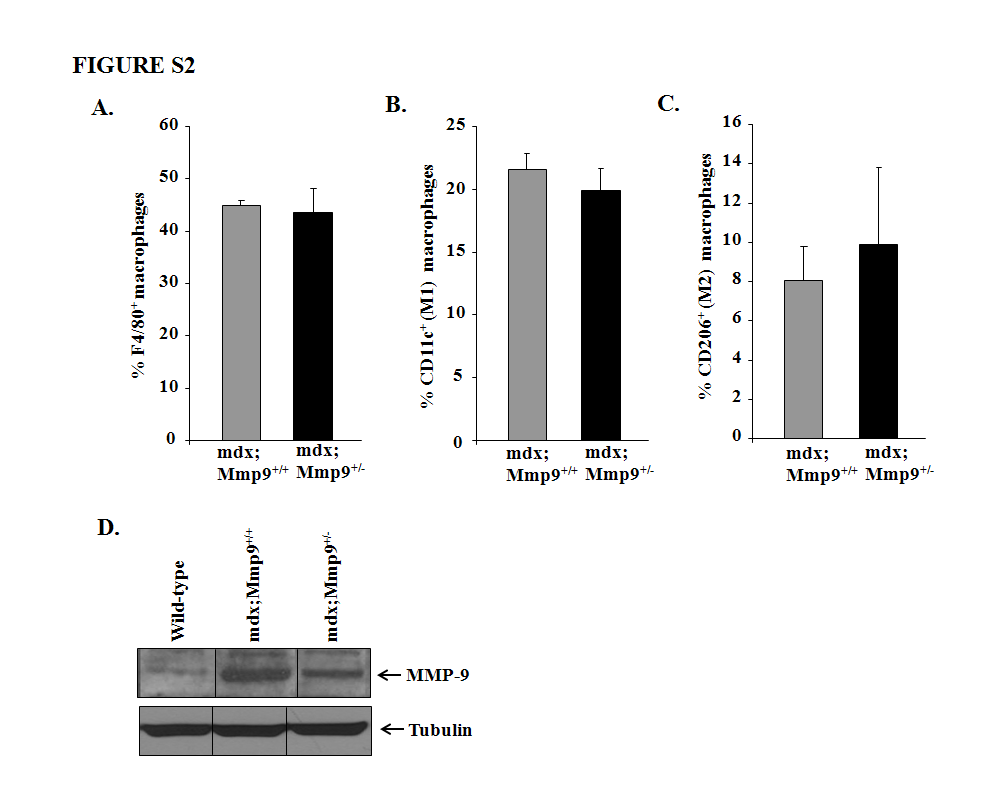

Supplement: Figure S2 — Effect of heterozygous deletion of Mmp9 gene on macrophage content and protein levels of MMP-9 in 4-week old mdx mice. GA muscle of 4-week old mdx;Mmp9+/+ and mdx;Mmp9+/− mice were isolated and processed for FACS analysis for F4/80+, CD11c+ (M1) and CD206+ (M2) macrophages after gating out CD45+ and Sca1+ cells. M1 and M2 macrophages were quantified in F4/80+ macrophage population. Bar diagrams presented here show (A) total F4/80+ macrophages, (B) M1 macrophages, and (C) M2 macrophages. Error bars represent SD. (D) Representative immunoblots showing MMP-9 and unrelated protein tubulin in GA muscle of 4-week old wild-type, mdx;Mmp9+/+, and mdx;Mmp9+/− mice. Black lines indicate that intervening lanes have been spliced out. (TIF) [file pone.0072121.s002.tif]
