# Supplementary material for: Matrix Metalloproteinase-9 Inhibition Improves Proliferation and Engraftment of Myogenic Cells in Dystrophic Muscle of mdx Mice
Source: PLoS One. 2013 Aug 15;8(8):e72121. doi: 10.1371/journal.pone.0072121 (PMC3744489; doi:10.1371/journal.pone.0072121)
Supplement: Table S1 — Sequence of the primers used for QRT-PCR assay. (DOCX) [file pone.0072121.s003.docx]

**Table S1**

| **Gene name** | **Forward primer (5’-3’)** | **Reverse primer (5’-3’)** |
| --- | --- | --- |
| Notch1 | CAGGAAAGAGGGCATCAG | AGCGTTAGGCAGAGCAAG |
| Notch2 | GCAGGAGCAGGAGGTGATAG | GCGTTTCTTGGACTCTCCAG |
| Notch3 | GTCCAGAGGCCAAGAGACTG | CAGAAGGAGGCCAGCATAAG |
| Dll1 | ACTGTACTCACCATAAGCCGTGCA | TCAGCTCACAGACCTTGCCATAGA |
| Dll4 | CACTTGCCACGATCTGGAGAAT | TGCCCACAAAGCCATAAGGA |
| Jagged1 | AACAAAGCTATCTGCCGACAGG | GGCTGATGAGTCCCACAGTAATTC |
| Jagged2 | TTGGTGGCAAGAACTGCTCAGT | GCTGTCACAGATGCAGGAGAAGTT |
| Hes1 | GCACAGAAAGTCATCAAAGCC | TTGATCTGGGTCATGCAGTTG |
| Hes6 | GCCGGATTTGGTGTCTACAT | TCCTGAGCTGTCTCCACCTT |
| HeyL | CAGATGCAAGCCCGGAAGAA | ACCAGAGGCATGGAGCATCT |
| Wnt3a | GCACCACCGTCAGCAACAG | GGGTGGCTTTGTCCAGAACA |
| Wnt4 | CTGGAGAAGTGTGGCTGTGA | GGACGTCCACAAAGGACTGT |
| Wnt5a | GGCATCAAGGAATGCCAGTA | GTACGTGAAGGCCGTCTCTC |
| Wnt7a | TGAAGAGGACCCAGTGACAGG | GGCGTACTGGTGTGTGTTGT |
| Wnt11 | GTAGGGCCTTCGCTGACAT | CGATGGTGTGACTGATGGTG |
| Frizzled1 | GCCGGCTGAGCTTGGAACTT | AACCAAAGCAGCAGCAGCAGC |
| Frizzled2 | CATCTCCATCCCGCTGTGCA | AGCACAGGAAGAAGCGCAGCTC |
| Frizzled4 | GGCTACAACGTGACCAAGATGCC | GCACATTGGCACATAAACCGAAC |
| Frizzled6 | GCGGCGTTTGCTTCGTT | CACAGAGGCAGAAGGACGAAGT |
| Axin2 | TTTGGCACAGCTAGAGGAAG | TGGCTCTTTGTGATCTTCTGG |
| TNF-α | GCATGATCCGCGACGTGGAA | AGATCCATGCCGTTGGCCAG |
| IL-1β | CTCCATGAGCTTTGTACAAGG | TGCTGATGTACCAGTTGGGG |
| IL-6 | CCTTCTTGGGACTGATGCTGG | GCCTCCGACTTGTGAAGTGGT |
| IFN-γ | GACAATCAGGCCATCAGCAAC | CGGATGAGCTCATTGAATGCTT |
| IL-4 | GGATGTGCCAAACGTCCTC | GAGTTCTTCTTCAAGCATGGAG |
| IL-10 | CAAGGAGCATTTGAATTCCC | GGCCTTGTAGACACCTTGGTC |
| CD163 | GCAAAAACTGGCAGTGGG | GTCAAAATCACAGACGGAGC |
| β-actin | CAGGCATTGCTGACAGGATG | TGCTGATCCACATCTGCTGG |
